# The effects of epithelial–mesenchymal transitions in COPD induced by cigarette smoke: an update

**DOI:** 10.1186/s12931-022-02153-z

**Published:** 2022-08-31

**Authors:** Xiaoshan Su, Weijing Wu, Zhixing Zhu, Xiaoping Lin, Yiming Zeng

**Affiliations:** Department of Pulmonary and Critical Care Medicine, The Second Affiliated Hospital of Fujian Medical University, Respirology Medicine Centre of Fujian Province, Quanzhou, China

**Keywords:** Epithelial–mesenchymal transition, Cigarette smoke, COPD, Signaling pathways

## Abstract

Cigarette smoke is a complex aerosol containing a large number of compounds with a variety of toxicity and carcinogenicity. Long-term exposure to cigarette smoke significantly increases the risk of a variety of diseases, including chronic obstructive pulmonary disease (COPD) and lung cancer. Epithelial–mesenchymal transition (EMT) is a unique biological process, that refers to epithelial cells losing their polarity and transforming into mobile mesenchymal cells, playing a crucial role in organ development, fibrosis, and cancer progression. Numerous recent studies have shown that EMT is an important pathophysiological process involved in airway fibrosis, airway remodeling, and malignant transformation of COPD. In this review, we summarized the effects of cigarette smoke on the development and progression of COPD and focus on the specific changes and underlying mechanisms of EMT in COPD induced by cigarette smoke. We spotlighted the signaling pathways involved in EMT induced by cigarette smoke and summarize the current research and treatment approaches for EMT in COPD, aiming to provide ideas for potential new treatment and research directions.

## Introduction

Chronic obstructive pulmonary disease (COPD) is a common, preventable, and treatable condition characterized by persistent respiratory symptoms and airflow restriction caused by respiratory and/or alveolar abnormalities, usually caused by high exposure to harmful particles or gases [1]. COPD is currently the third leading cause of death globally and is associated with significant social and economic burdens [2, 3].

The most commonly encountered risk factor for COPD is long-term direct or passive exposure to cigarette smoke (CS). Cigarette smoke contains several toxic compounds that contribute to the pathogenesis of many respiratory diseases, such as COPD and lung cancer [4]. Compared with non-smokers, smokers are more susceptible to suffering from respiratory symptoms and abnormal lung function, with a higher annual decline rate of FEV1 and a higher COPD mortality rate [5, 6].

Epithelial–mesenchymal transformation (EMT) is a unique biological process in which epithelial cells lose their polarity and transform into mobile mesenchymal cells [7]. EMT serves a crucial role in embryonic development, chronic inflammation, tissue reconstruction, cancer metastasis, and a variety of fibrotic diseases [8, 9]. Moreover, EMT is increasingly being considered a possible core pathophysiological factor in COPD and lung cancer progression [10–14]. In smoking-related COPD, recent studies further highlight that EMT is associated with airway remodeling, airway fibrosis, and subsequent airflow obstruction and may be associated with a higher prevalence of lung cancer [15–18]. All of these findings suggest the importance of EMT in the development of smoking-related COPD. It is important to better understand the mechanisms that lead to EMT and to develop treatments that target EMT.

Here, we provide a brief overview of the pathogenesis of cigarette smoke-induced COPD. We focus on the specific changes and underlying mechanisms of EMT in COPD induced by cigarette smoke, which may provide new ideas for innovative prevention or treatment targets for COPD. This review may contribute not only to understanding the key elements of EMT but also to developing new strategies for the treatment of COPD.

## Cigarette smoke and COPD

### Cigarette smoke

Cigarette smoke is a complex aerosol composed of gas phase and particle phase. It contains more than 7000 different types of chemical components with various toxic and carcinogenic properties [19]. Among these substances are nicotine, carbon monoxide, carbon dioxide, tar, ammonia, formaldehyde, acrolein, acetone, polycyclic aromatic hydrocarbons (benzo (a) pyrene), hydroxyquinones, nitrogen oxides, and heavy metals (nickel, cadmium, chromium, and arsenic) [20]. The adverse effects of cigarette smoking on human health have been well documented over the past decades. It has been suggested that cigarette smoking has irreversible effects on genetic material (DNA mutations) as well as possibly reversible effects on the epigenetic landscape (DNA methylation and chromatin modification) [21]. It is increasingly recognized that smoking not only causes health problems for smokers and passive smokers, but also environmental hazards, with consequences for ecosystems and human health [22]. Cigarette smoke is an important risk factor for several diseases, such as COPD, cardiovascular disease, and cancer [23–25]. Smoking is the primary cause of preventable disease globally. Public health should promote understanding of the current pathology of smoking-related diseases and encourage individuals to reduce their exposure to cigarette smoke, thereby reducing the harmful consequences of related diseases.

### Effects of cigarette smoke on the development and progression of COPD

Factors that influence the development and progression of COPD include genetic factors, age and gender, and exposure to particulate matter such as cigarette smoke. Cigarette smoking is the most crucial risk factor for the development of COPD. Repeated cigarette smoke exposure can cause chronic inflammation in the lungs, which increases the number of certain inflammatory cells, as well as structural changes resulting from repeated damage and repair. These changes contribute to the clinical features of COPD, including airway remodeling, chronic bronchitis, and emphysema [1]. The following is a brief overview of the pathological changes, and cellular and molecular mechanisms underlying smoking-induced COPD.

The ciliated epithelium of the respiratory tract is the first protective line against harmful substances. It removes pathogens from the mucus layer through mucociliary clearance, establishes barriers through tight and adherent junctions, and activates and recruits immune cells in the submucosa through cytokine and chemokine production [26, 27]. Cigarette smoke contains a large number of toxic substances, including a large number of oxygen metabolite-derived or reactive oxygen species (ROS), which can directly disrupt this physical barrier, resulting in increased permeability of respiratory epithelial cells and hindering clearance of mucus cilia [28]. Importantly, cigarette smoke could induce oxidative damage to cell membrane lipids through various mechanisms, such as DNA damage, lipid peroxidation, amino acid oxidation, inorganic enzyme cofactor oxidation, etc. [29–31].

**Fig. 1:**
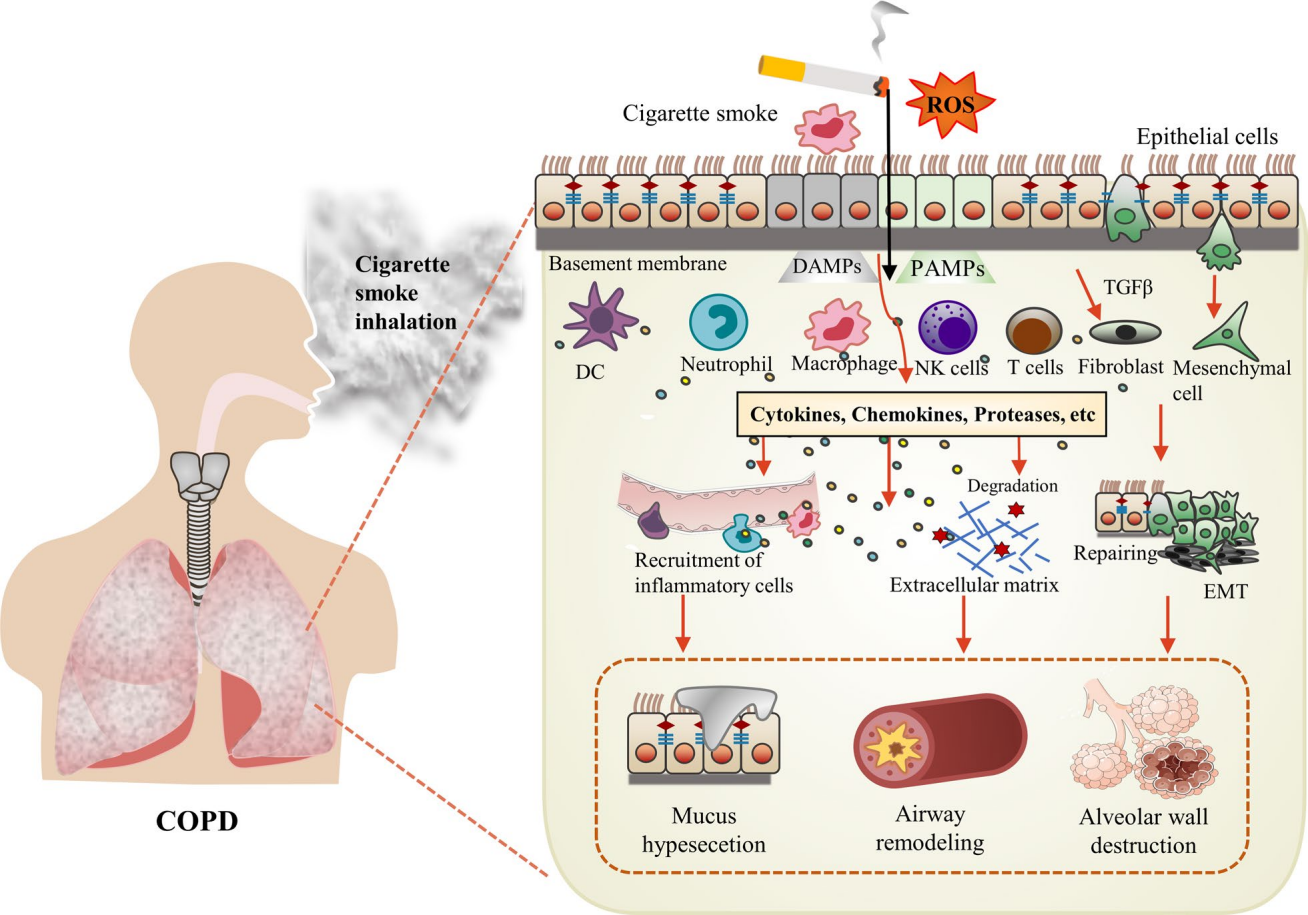
Overview of the effect of cigarette smoke on the development and progression of COPD. Cigarette smoke activates DAMP and PAMPS in lung epithelial cells and alveolar macrophages and produces excess ROS. Stimulants such as ROS attract macrophages, neutrophils, DC, NK cells, and T lymphocytes to migrate to the lungs by releasing cytokines and chemokines (e.g., NF-κB, IL-8, IL-1β, ROS, TNF-α). During the chronic phase, inflammatory cells are continuously recruited and release inflammatory mediators, such as proteases (e.g., MMPs and neutrophil elastase), chemokines, cytokines, and ROS. Epithelial cells and macrophages also release fibroblast mediators, such as TGFβ, which activate fibroblasts and lead to small airway fibrosis. In addition, cigarette smoke induces epithelial EMT, which leads to dysfunction of endothelial cells and epithelial barrier, hinders tissue repair, and ultimately leads to fibrosis. Hence, long-term exposure to cigarette smoke causes sustained inflammation and oxidative stress in the lungs, leading to repeated repair and remodeling (leading to airway remodeling), degrading the alveolar walls (leading to emphysema), and stimulating mucus hypersecretion (leading to chronic bronchitis)

In the early stage of smoking, protective responses and DNA repair triggered by the lung barrier may inhibit these changes to some extent [32]. In the case of long-term smoking, these mechanisms seem to break down and lead to disease progression. Chronic inflammation and the oxidant–antioxidant balance are the main driving molecular mechanisms promoting the progression of COPD and exacerbations [33, 34]. Specifically, cigarette smoke activates damage-associated molecular patterns (DAMP) and pathogen-associated molecular patterns (PAMPS) in lung epithelial cells and alveolar macrophages, which activate Toll‑like receptors (TLRs) and NOD‑like receptors (NLRs). This process produces excess ROS and reactive nitric oxide (RNS), which may lead to an oxidation/antioxidant imbalance [35, 36]. Stimulants such as ROS in cigarette smoke attract macrophages, neutrophils, dendritic cells, natural killer cells, and T lymphocytes to migrate to the lungs by releasing cytokines and chemokines (e.g., NF-κB, IL-8, IL-1β, ROS, and TNF-α). During the chronic phase, inflammatory cells are continuously recruited and release inflammatory mediators, such as proteases (e.g., MMPs and neutrophil elastase), chemokines, cytokines, and ROS. Epithelial cells and macrophages also release fibroblast mediators, such as TGFβ, which activate fibroblasts and lead to small airway fibrosis. In addition, cigarette smoke impairs structural cell function and initiates the EMT, a process leading to dysfunction in endothelial as well as an epithelial barrier, hamper tissue repair, and eventually leading to fibrosis. Thus, chronic exposure to cigarette smoke causes persistent inflammation and oxidative stress in the lungs, leading to repeated repair and remodeling (leading to airway remodeling), stimulating mucus hypersecretion (leading to chronic bronchitis), and degrading the alveolar walls (leading to emphysema) (Fig. 1) [28, 37–40]. Cigarette smoke induces COPD-related airway remodeling phenotypes, including airway epithelial hyperplasia, myocyte hyperplasia, squamous metaplasia, EMT, ciliary alterations, loss of secretory cells that produce Scgb1a1, and reduced integrity of the apical junction barrier that controls airway epithelial permeability. Together, these phenotypes lead to airway obstruction and reduced airway epithelial barrier and host defense function [28, 41].

In recent years, it has become increasingly clear that the structural integrity and functional stability of multiple organelles are important for the function and survival of cells. Various organelle dysfunction plays an important role in the pathogenesis and progression of COPD [42]. In fact, these organelles showed significant structural derangement and aberrant function under exposure to cigarette smoke. The excessive oxidative burden is considered to be one of the underlying mechanisms of COPD epithelial barrier breakdown. Chronic exposure of the lungs to cigarette smoke disrupts the mitochondrial activity and endoplasmic reticulum (ER) homeostasis that triggers an unresolvable unfolded protein response activation. Mitochondrial dysfunction induces oxidative stress through excessive production of mitochondrial ROS, thereby increasing epithelial barrier permeability. Furthermore, cigarette smoke leads to the accumulation of damaged and misfolded proteins in the endoplasmic reticulum (ER), a condition known as ER stress, accompanied by enhanced unfolded protein response (UPR). Although UPR is a compensatory cellular response that reduces protein synthesis and enhances protein folding and degradation, UPR also contributes to lung cell apoptosis and lung inflammation during excessive ER stress [43–47]. Therefore, quality control of multiple organelles is of great significance for maintaining cell survival and function, and maybe a potential therapeutic target for COPD.

### The possible link between cigarette smoking-related COPD and lung cancer

Cigarette smoking is the principal factor driving the pathogenesis and progression of COPD and lung cancer. Several epidemiological and observational cohort studies have systematically confirmed the close relationship between COPD and lung cancer [48–52]. Epidemiological studies have shown a four- to sixfold increased risk of lung cancer in patients with COPD, with the onset of lung cancer associated with the severity of COPD [53, 54]. Significantly, many phenotypic and genotypic features of COPD, such as a history of smoking, chronic bronchitis, airway obstruction, and emphysema, are associated with an increased risk of lung cancer [55, 56]. COPD has been reported to be an additional burden and risk factor for the development of lung cancer, primarily squamous cell carcinoma, especially in smokers [10, 57]. Genetic susceptibility, DNA methylation changes, local chronic lung inflammation, and abnormal repair mechanisms in COPD patients are important potential factors for lung cancer development [58–60]. Indeed, COPD and lung cancer have many common biological mechanisms, including chronic inflammation, oxidative stress, matrix degradation, genetic susceptibility, lung barrier dysfunction, and epithelial–mesenchymal transition (EMT) [4, 59, 61]. Among these, oxidative stress, chronic inflammation, and EMT are the most studied drivers of carcinogenesis. Cigarette smoke exposure causes inflammation, oxidative stress, and lung barrier dysfunction, and leads to EMT that end up with ultimately abnormal tissue repair [4]. The protracted inflammation gave rise to EMT which ended up with aberrant tissue repair. This might help explain the link between COPD and lung cancer in smokers and may provide guidance for management and prevention strategies for COPD and lung cancer.

## Epithelial–mesenchymal transformation in chronic obstructive pulmonary disease

### Epithelial–mesenchymal transformation

The pulmonary epithelial cell lining forms an external protective barrier against the environmental toxins produced by smog and microbial infection. EMT is a unique biological process, that refers to epithelial cells losing their polarity and transforming into mobile mesenchymal cells. Generally, EMT can be classified into three main types based on the physiological tissue setting: type I EMT occurs in embryonic development and organogenesis, type II EMT occurs in tissue repair and fibrosis, and type III EMT occurs in epithelial malignancies associated with aggressive or metastatic phenotypes [62]. The Morphological alterations characteristic of EMT include the disruption of epithelial cell junctions, the destruction of polar complex, and the reorganization of cytoskeletal structure. Molecularly, EMT is characterized by downregulation of epithelial junction proteins (e.g., E-Cadherin and Occludins) and activation of EMT transcriptional activators (e.g., Snail, Slug, and Twist) and mesenchymal markers (e.g., S100A4, Vimentin, Fibronectin, and N-Cadherin) [63].

### Alterations of epithelial–mesenchymal transformation biomarkers in COPD

The role of EMT is well documented in embryonic development, wound healing, tumor progression, and tissue fibrosis [64]. Aberrant wound repair and fibrosis are associated with many respiratory diseases. EMT has been implicated as fundamental for lung development and many respiratory diseases, particularly those characterized by increased deposition of collagen and other ECM proteins in the airways or parenchyma. These diseases include COPD, lung cancer, asthma, pulmonary fibrosis, and bronchiolitis obliterans syndrome [62, 65]. Extensive research has shown that EMT is activated in the airway tissue of smokers, particularly in those current-smoking COPD patients [66–69]. EMT is increasingly being considered a possible core pathophysiological factor in COPD and lung cancer progression [10–14]. Interestingly, EMT has been linked to airway fibrosis, airway remodeling, and airflow obstruction, and may even contribute to the high incidence of lung cancer in COPD patients [8, 12, 70]. In this part, we will describe in detail the alterations of EMT biomarkers in COPD.

EMT transcription factors and mesenchymal markers were up-regulated and epithelial markers down-regulated in COPD, and were associated with lung function, as shown in Table 1. Specifically, the expression of EMT-related transcription factor Snail1 in α 1-antitrypsin deficient COPD was significantly higher than that in normal COPD [71]. Moreover, Mahmood et al. [17] found that transcriptional factors Snail1 and Twist were upregulation and nuclear translocation in smokers and current-smoking COPD, and their expression is closely associated with EMT activity (S100A4 expression) and the levels of airflow obstruction.

**Table 1:** Alterations in epithelial–mesenchymal transitions biomarkers in COPD and pathological significance

EMT biomarkers	Physiological role	Alterations in COPD	Refs
Snail1	EMT transcriptional activator	Higher expression in smokers, COPD with current smoking, and COPD with α1-antitrypsin deficiency, and is associated with EMT activity and lung function	[17, 71]
Twist	EMT transcriptional activator	Upregulation and nuclear transport in smokers and current-smoking COPD, and expression is closely related to both emt activity and airway obstruction	[17, 69]
S100a4	Mesenchymal marker	Upregulation in COPD and inversely associated with airflow limitation	[67, 69, 81, 82]
Zo-1	Tight junction marker	Deceased in the smokers and patients with COPD	[68, 74]
E-cadherin	Epithelial marker	The lower expression was found in smokers and patients with COPD	[68, 74–78]
N-cadherin	Cell-surface proteins	Increased in smokers and COPD	[75, 78]
Collagen type I	ECM proteins	Higher expression in smokers and COPD	[68, 75]
Vimentin	Mesenchymal markers	Increased in smokers and COPD, epithelial expression of vimentin correlated with airway obstruction	[67, 68, 74, 75, 77, 78]
Α-SMA	Cytoskeletal marker	Increased in smokers and COPD	[68, 74, 75, 78]
Fibronectin	ECM proteins	Increased in smokers and COPD	[75]
Mmp9	Basement membrane	Increased in smokers and COPD	[75]
Β2-microglobulin	MHC I light chain	Increased in COPD	[88, 89]
Sphingosine-1-phosphate	Bioactive sphingolipid metabolite	Upregulated and inversely associated with lung function in COPD	[81]
Cullin4A	E3 ubiquitin ligase	Upregulation in smokers and COPD, and negatively correlated with the FEV1%	[97]

Among the expression alterations of epithelial and mesenchymal cell markers, E-cadherin is considered a prominent hallmark of EMT and serves a central role in the EMT process. E-cadherin is an adhesion molecule responsible for the organization of interepithelial junctions [72, 73]. Numerous studies have shown that E-cadherin was markedly decreased in smokers and COPD [68, 74–78]. Particularly, the expression of E-cadherin was positively related to FEV1/VC ratio [74]. The extracellular portion of E-cadherin can be degraded by proteases including MMPs to form circulating molecules soluble E-cadherin (sE-cadherin). Shirahata et al. [79] demonstrated that Plasma sE-cadherin levels were significantly lower in patients with COPD and symptomatic smokers than in healthy smokers and healthy non-smokers, and sE-cadherin levels were associated with the severity of airflow limitation in COPD and symptomatic smokers plasma.

In addition, mesenchymal markers (S100A4, N-cadherin, Vimentin, and α-SMA proteins) and ECM proteins (type I collagen and fibronectin) were also found increased in smokers and COPD. S100A4 (also named fibroblast specific protein 1, FSP1) is considered a canonical mesenchymal marker in EMT with biological functions of promoting cell motility, invasion, ECM remodeling, autophagy, and angiogenesis [80]. Numerous studies have demonstrated that S100A4 was upregulated in COPD and inversely associated with airflow limitation [67, 69, 81, 82]. Vimentin is expressed in all mesenchymal cells and is the core of EMT-mediated metastasis. During EMT, vimentin can induce cell migration by forming cell processes, reducing cell adhesion, and increasing cell migration ability [83]. Vimentin has also been found to be upregulated in smokers and COPD [67, 68, 74, 75, 77, 78], and the expression of vimentin in the bronchial epithelium of COPD is associated with basement membrane thickening and airflow limitation [74].

Additionally, there is an increasing number of studies identifying new biomarkers for EMT in COPD. β2-microglobulin (β2M), also known as the class I major histocompatibility complex (MHC I) light chain, is involved in the regulation of EMT processes in several diseases [84–87]. It was shown that plasma β2M concentrations were significantly higher in smoking patients with COPD and emphysema than those in normal subjects [88]. Wu et al. [89] indicated that β2M was increased in COPD patients and was correlated with lower pulmonary diffusing capacity values, increased alveolar wall/septal thickening (fibrosis changes), and higher expressions of TGF-β1, Smad4, and a-SMA. They further found that β2M expression of lung tissues was correlated with EMT and fibrosis progression in cigarette smoke-exposed COPD rats. Sphingosine-1-phosphate (S1P), a bioactive sphingolipid metabolite, plays an important role in the occurrence and development of cancer by regulating and promoting cell growth, migration, invasion, and cell survival [90]. Previous studies found that S1P drove the EMT process via the TGF-β axis and was correlated with lung function in patients with idiopathic pulmonary fibrosis and asthma-like disease [91, 92]. In vivo studies have shown that S1P expression is increased and associated with pulmonary resistance in cigarette smoke-induced COPD mice [93]. The latest finding showed that serum S1P was upregulated and inversely associated with lung function in patients with COPD. In addition, serum S1P was positively associated with mesenchymal marker S100A4 in COPD [81]. Cullin 4A (CUL4A), an E3 ubiquitin ligase, is involved in the regulation of the cell cycle, DNA replication, and DNA damage repair [94]. CUL4A has highly expressed in non-small cell lung cancer (NSCLC) tissues and can promote lung cancer progression by inducing EMT [95, 96]. Ren et al. [97] found that the expression of CUL4A in lung epithelium of smokers and smoke patients with COPD was significantly higher than that of non-smokers and CUL4A was negatively correlated with the FEV1%. Moreover, they found that silencing CUL4A inhibited CSE-induced EMT in human small airway epithelial cells.

During the EMT process in COPD, alterations in cell phenotypes contributed to the emergence of specific biomarkers. These specific biomarkers can identify levels of EMT activity level and may be used to assess airflow restriction, COPD exacerbation risk, and malignant transformation. Future studies are needed to clarify the association of specific EMT biomarkers with lung function, pulmonary fibrosis, and malignant transformation in smoking-related COPD patients.

## Signalling pathways involved in EMT induced by cigarette smoke in COPD

**Fig. 2:**
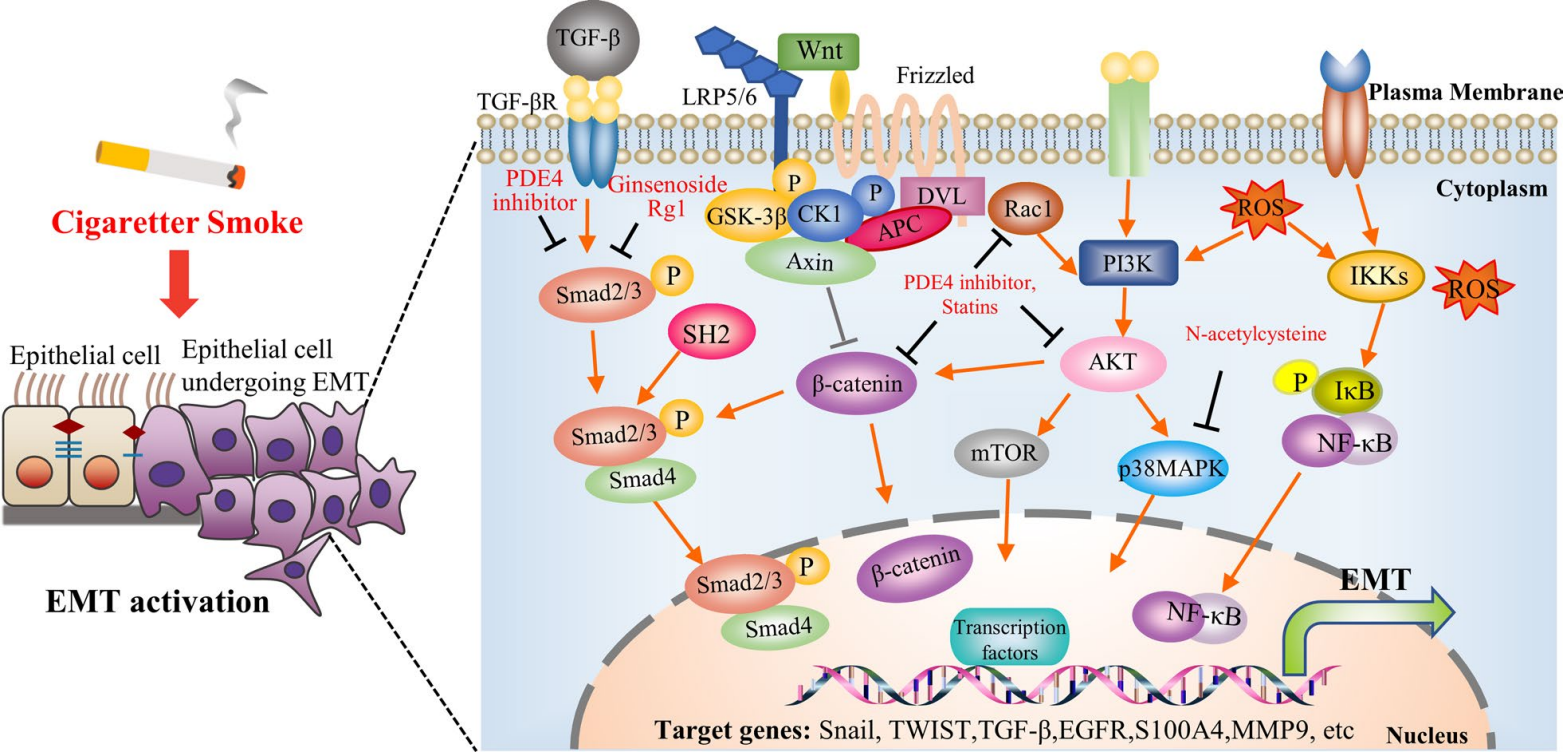
Schematic overview of EMT-related signaling pathways in cigarette smoke-induced COPD and the potential therapies targeting these signaling pathways. Cigarette smoke can induce EMT in COPD through multiple different signaling pathways. These pathways are intricate and inextricably partly crosslinked. Additionally, potential therapies based on targeting these signaling pathways were shown. Each is depicted in the text

Recently, several studies have explored the important role of cigarette smoke in the induction of EMT in COPD. We summarized these reports and suggested that cigarette smoking may induce the occurrence of EMT by using the specificity of different signaling pathways. This section provides a brief overview of the mechanisms and signaling pathways involved in EMT induced by cigarette smoke in COPD. These signaling pathways include the TGF-β/Smad signaling pathway, the Wnt/β-catenin signaling pathway, PI3K-Akt signaling pathway, and NF-κB signaling pathway, etc. (Fig. 2). A better understanding of the signaling pathways underlying cigarette smoke-induced EMT could provide new targets and potential strategies for COPD patients.

### TGF-β/Smad signaling pathway

The transforming growth factor-β (TGF-β) superfamily consists of a group of distinct polypeptides involved in regulating a wide range of cellular and physiological processes, including proliferation, differentiation, migration, adhesion, ECM synthesis and cell death [98]. TGF-β can induce EMT via both canonical TGF-β/Smad signaling pathway and non-canonical pathway (e.g. ERK, P38, MAPK, PI3K/Akt) [99, 100]. In the canonical TGF-β/Smad signaling pathway, TGF-β ligands regulate key transcription factors that promote EMT through Smads protein and synergistic kinase pathway. In general, TGF-β1 and the Smad signaling pathways are regarded as the key to the EMT-related pathogenesis of COPD [101, 102]. Particularly, TGF-β1 is a multifunctional cytokine that induces angiogenesis and regulation of extracellular matrix (ECM) components, a which is considered to be a key regulator of COPD airway pathology [16]. Studies have shown that exposure to TGF-β1 induces mesenchymal phenotype and EMT in cultured human bronchial and lung epithelial cells in vitro [74, 103–105]. In addition, TGF-β1 expression was significantly increased in the airway epithelium of both smokers and COPD patients, accompanied by the expression of ρ Smad2/3 [16, 106]. Likewise, cigarette smoke induces EMT in lung and bronchial epithelial cells through TGF-β1/Smad signaling pathway in vivo and in vitro [107–111]. Together, these studies suggest that cigarette smoke targeting the TGF-β/Smad signaling pathway induces EMT in COPD.

### Wnt/β-catenin signaling pathway

The Wnt/β-catenin signaling pathway plays an important role in embryonic development, adult tissue homeostasis, and regeneration, and its abnormal regulation is closely related to various diseases [112]. After activation of the Wnt/β-catenin pathway, β-catenin is translocated to the nucleus and interacts with the transcription factor LEF/TCF to activate transcription. In addition, the Wnt/β-catenin signaling pathway has been reported to regulate EMT in several cancers [113]. Interestingly, β-catenin is a crucial regulator in the Wnt/β-catenin signaling pathway, and phosphorylation or down-regulation of β-catenin can inhibit the activation of the Wnt/β-catenin signaling pathway [114]. Numerous recent studies indicated that the Wnt/β-catenin signaling is activated in smokers and COPD, and is strongly correlated with EMT activity and airway obstruction. Carlier et al. [66] reported that Wnt/β-catenin signaling pathway-related genes and proteins were significantly upregulated in the airway epithelium of COPD smokers. They further found that activation of the Wnt/β-catenin signaling pathway in human airway epithelial cells from COPD smoking patients resulted in increased Vimentin expression, increased fibronectin release, and enhanced TGF-β1/Smad signaling. Conversely, inhibition of the Wnt/β-catenin signaling pathway increases ciliary cell count, epithelial polarity, and barrier function, while inhibiting EMT, thereby reversing COPD characteristics. This study demonstrates the relationship between Wnt/β-catenin and EMT and its important role in promoting COPD pathology. Moreover, the expression of β-catenin and Snail1 is up-regulated in the airway wall of both smokers and COPD, and their expression was strongly associated with typical EMT biomarkers (S100A4) and airway obstruction [17, 115]. Likewise, in vitro studies have shown that cigarette smoking and nicotine induce EMT by activating the Wnt/β-catenin signaling pathway in HBE cells and A549 cells [116, 117]. Therefore, regulation of Wnt/β-catenin could serve as a promising therapeutic strategy to control EMT induction by cigarette smoking in COPD.

### PI3K/AKT signaling pathway

The PI3K/AKT signaling pathway is a key regulator of important biological functions, including metabolism, cell proliferation, epithelial–mesenchymal transition, survival, and apoptosis [118]. Rac1 is involved in several dynamic cell biological processes, such as cell survival, cell–cell contact, cell motility, EMT, and cell invasion [119]. A previous study showed CS-induced EMT via Rac1/PI3K/Akt and Rac1/Smad2 signaling pathways. Pharmacological inhibition of Rac1 could alleviate TGF-β1 production and prevent alterations in the expression of EMT markers in CS-exposed mice. In addition, knockdown or inhibition of Rac1 ameliorated CSE-induced TGF-β1 release and CSE-induced EMT and inhibited CSE-induced Akt and Smad2 activation in A549 pulmonary epithelial cells. Furthermore, inhibition of PI3K, Akt, or Smad2 could suppress CSE-induced alterations in epithelial and mesenchymal marker expression [107]. Another study has found that Rac1 could regulate cigarette smoke-induced pulmonary inflammation in the lung through the STAT3 and Erk1/2 MAPK signaling pathways [120]. According to Milara et al. [121], in primary human bronchial epithelial cells, CSE induces EMT partially through the activation of Rac1, PI3K/Akt/β-catenin pathways, and the generation of ROS. Particularly, EMT can be induced by TGF-β through the activation of PI3K/AKT and MAPK in the Smad-independent pathway [122–124]. mTOR is a member of the PI3K family and an effector protein downstream of the PI3K/AKT signaling pathway [125]. A study by Jiang et al. [126] found that CS exposure in mice and CSE exposure in bronchial epithelial cells could induce EMT by activating the Akt signaling pathway. They further demonstrated inhibition of Akt activity can inhibit the progression of smoke-induced EMT by down-regulating TGF-β1/Akt/Smad/mTOR and Akt/P38 MAPK signaling pathway. Collectively, these results suggest that PI3K/AKT signaling pathway activation may be involved in the pathogenesis of CS-induced pulmonary EMT and has potential therapeutic significance in COPD, lung cancer, and other smoking-related diseases.

### NF-κB signaling pathways

The transcription factor nuclear factor-kappa B (NF-kB) is an essential stressor in the cellular environment and regulates a series of genes involved in survival, oxidative stress, inflammation, and immunity [127]. EMT is regarded as an intersection of inflammation, oxidative stress, fibrotic diseases, and cancer. Moreover, NF-kB is a major pro-inflammatory transcription factor activated by inflammatory cytokines and ROS, which is one of the key roles in the formation of EMT [128, 129]. Zhao et al. [130] reported that the NF-κB signaling pathway is involved in CSE-induced EMT in HBE cells, suggesting that NF-κB activation acts as a bridge between CSE-induced chronic inflammation, EMT, and lung cancer. The data showed that inhibition of NF-κB activation could block CSE-induced upregulation expression of E-cadherin, and reverse the downregulation of IL-6 and N-cadherin in HBE cells. In addition, the study found that silencing of NF-κB decreased CSE-induced colony formation and the invasion and migration capacities in HBE cells [131]. Li et al. [132] further demonstrated that CSE can induce EMT through activation of the IL17R/NF-κB signaling pathway in murine bronchial epithelial cells. Interestingly, Hong et al. [133] used *N*-acetylcysteine (NAC), an inhibitor of the oxidative stress signaling pathway, to prove that the oxidative stress signaling pathway is involved in the cigarette smoke-induced EMT process. Taken together, the NF-κB signaling pathway is a suitable therapeutic target for CSE-induced EMT, inflammation, oxidative stress, and malignant transformation in COPD.

### Other signaling pathways

EMT is an extremely complex pathological process, which is often not the activation of a single signaling pathway. The protein tyrosine phosphatase Shp2 is thought to be involved in chronic pneumonia and fibrosis [134]. Shp2 plays a key role in acute cigarette smoke-induced lung inflammation, in which pulmonary epithelial knockout of Shp2 reduced IL-8 release and lung inflammation in CS-exposed mice [135]. Furthermore, Liu et al. [136] found that Shp2 inhibition reduced BMP-9 production, EMT progression, and phosphorylation of ERK1/2, JNK, and SMAD2/3 in CS and CSE exposure mouse lungs and pulmonary epithelial cells. MAPK, an intracellular Ser/Thr protein kinase, is involved in a variety of signaling pathways and plays an important role in cell cycle regulation [137]. Studies have reported that NAC can improve COPD-related pulmonary fibrosis by activating immune response and suppressing the EMT process through VWF/P38 MAPK signaling pathway in vivo and in vitro experiments [15]. In addition, TACE/TGF-α/EGFR signaling pathway is found activated in CSE-exposed human airway epithelial cells, and blocking the TACE/TGF-α/EGFR signaling pathway can inhibit the CSE-induced EMT process [138].

P53 (TP53), as a tumor suppressor, is thought to be the most frequently mutated gene in cancer cells. There is increasing evidence that mutated p53 enhances tumor metastasis and affects EMT processes [139]. Moreover, a large number of recent studies have shown that p53 signaling plays a role in regulating the EMT process in lung cancer [140–143]. Notably, chronic exposure to cigarette smoke has been linked to the development of p53 mutations and may contribute to p53 mutations in lung cancer [144–147]. A bioinformatics study has found that the P53 pathway may play an important role in promoting the progression of COPD to lung squamous cell carcinoma [148]. In addition, studies have shown that p53 gene polymorphisms are associated with apoptotic signaling and smoking-related emphysema in smokers [149]. Additionally, previous studies have shown that p53 gene polymorphism was significantly related to the incidence of smoking-related COPD, and p53 protein was markedly increased in COPD smokers [150, 151]. Given the key role of p53 in EMT and lung cancer, cigarette smoke-induced EMT may involve p53; thus, the relationship between P53, EMT, and cancer transformation in cigarette smoking-related COPD requires further investigation.

In fact, EMT induced by cigarette smoke in COPD is a complex network involving the regulation of multiple signaling pathways. These pathways are intricate, inevitably partially cross-linked and require further exploration. Therefore, cigarette smoking induces EMT through the above signaling pathways, and whether there are other signaling pathways affecting EMT deserves further study.

## Potential therapies for EMT in COPD

At present, bronchodilators (short-acting Beta2-agonists (SABA) and long-acting Beta2-agonists (LABA)), antimuscarinic drugs (short-acting antimuscarinics (SAMAs), and Long-acting muscarinic antagonists (LAMAs)), methylxanthines (Theophylline), anti-inflammatory agents, Inhaled corticosteroids (ICS), antibiotics, mucolytic (mucokinetics and dmucoregulators), antioxidant agents (*N*-acetylcysteine, carbocysteine, and erdosteine) and Phosphodiesterase-4 (PDE4) inhibitors are commonly used drugs for the treatment of COPD [1]. However, many patients are unable to manage their daily symptoms even with standard care. Therefore, there is a critical need to explore new drug targets and develop more effective drugs. EMT plays a critical role in the development of smoking-related COPD airway remodeling disease and related lung cancer, especially in smoking-related COPD, where it may lead to airway remodeling, obstruction/occlusion, and tumor development. Therefore, drugs that inhibit the EMT process or kill EMT-type cells will be a novel therapeutic target for COPD. However, there are few studies on the anti-EMT effects of different drugs and even fewer data from human clinical studies in vivo. Drugs already used to target COPD may be able to retarget their long-term anti-EMT prophylaxis.

EMT is considered to be part of the pathophysiology of fibrosis, remodeling, and malignant consequences associated with COPD, and thus clinical trials targeting EMT have been conducted. The following sections, summarized in Table 2, will give an overview of EMT-targeting therapies and an understanding of how these may specifically or non-specifically target the EMT process. These therapies are Inhaled corticosteroids (ICS), *N*-acetylcysteine (NAC), Phosphodiesterase-4 (PDE4) inhibitors and statins, urokinase-type plasminogen activators and urokinase-type plasminogen activator receptor (uPA and uPAR), Adipose-derived stem cell-conditioned medium (ADSC-CM) and Ginsenoside Rg1. Partial potential therapies based on these signaling pathways are shown in Fig. 2. These therapies will require further mechanism exploration, and preclinical and clinical trials to establish their efficacy as a single or combined treatment of EMT in COPD.

**Table 2:** Summary of EMT-targeting therapy studies in COPD

Therapy	Refs	Study description	Main findings
Inhaled corticosteroids (ICS)	[152]	Phase IIa Randomized Controlled Trial. 34 COPD participants were randomized 2∶1 to fluticasone propionate 500 µg bid or placebo bid for 6 months	Treatment inhibited epithelial activation (EGFR expression), “clefts/fragmentation” in the Rbm, and EMT biomarkers (S100A4 and MMP-9)
	[156]	Nine prospective cohort studies included 181,859 COPD patients	ICS was associated with a decreased risk of lung cancer in patients with COPD
*N*-acetylcysteine (NAC)	[15]	Rat model. Except for the normal group of Rats in the COPD, the group was administered intratracheally lipopolysaccharide and then exposed to smoke for 30 min a day for 28 days	NAC reduced collagen volume fraction, α-SMA level, wall area/total bronchiole area, and the wall thickness/bronchiole diameter in COPD rat
Phosphodiesterase-4 (PDE4) inhibitors	[161]	Mouse model. Mice in the Bleomycin group were anesthetized and administered intratracheally bleomycin. Mice in the control group received an identical volume of intratracheal saline	Roflumilast reduced bleomycin-induced lung alpha(I)collagen transcripts, fibrosis, and vascular remodeling response in mice
	[162]	Mouse model. Chronic exposure mice were exposed to the smoke of three cigarettes/day for 5 days/week for 7 months. Control mice were exposed to room air	Roflumilast ameliorated cigarette smoke-induced lung inflammation and emphysema
	[163]	In vitro. Isolated HBECs from non-smokers, smokers, and COPD patients	RNO inhibited CSE induced the upregulation of α-SMA, vimentin, and collagen type I, and reversed the downregulation of E-cadherin, ZO-1, and KRT5 in HBEC. Moreover, RNO decreased a-SMA, vimentin, and collagen type I yet increased E-cadherin and ZO-1 in HBECs isolated from smokers and COPD patients
PDE4 inhibitors and statins	[121]	In vitro. Isolated HBEC from human lung tissue of patients undergoing surgery for lung cancer	RNO partly alleviates the CSE-induced EMT in WD-HBEC in vitro, and simvastatin increases the ability of RNO to inhibit CSE-induced EMT
uPA and uPAR	[174, 175]	In vitro. Human small airway epithelial cell lines (HSAEpiCs)	uPA and uPAR inhibition could block CSE-induced EMT by reversing E-cadherin and α-catenin expression and retarding the induction of N-cadherin and vimentin
ADSC-CM	[178]	In vitro. A549 cells	ADSC-CM culture could reverse CSE-induced decreased E-cadherin expression, increased vimentin expression, and accelerated cell migration in A549 cells
Ginsenoside Rg1	[108]	Mouse model and Human bronchial epithelial (HBE) cells line. Rats with COPD were exposed to the smoke of 3 cigarettes/day, 6 times per day, 6 days a week, for 12 weeks. The normal control group was exposed to room air	Ginsenoside Rg1 alleviated CS or CSE-induced EMT via blocking the regulation of α-SMA and E-cadherin expression by CS or CSE

### Inhaled corticosteroids (ICS)

Inhaled corticosteroids (ICS) have become a standard treatment in more severe COPD, based on empirical results from large multicenter studies. A “proof of concept” randomized controlled trial concluded that the use of inhaled corticosteroids for more than 6 months inhibited EMT-related changes in COPD patients. Results showed that epithelial activation (EGFR expression), “clefts/fragmentation” in the Rbm, and EMT biomarkers (S100A4 and MMP-9) were significantly regressed after treatment in the ICS group compared to the placebo group [152]. According to some cohort studies, inhaled corticosteroids may reduce the incidence of lung cancer [153–155]. Interestingly, EMT activity in epithelial cells may be prone to malignant transformation, and EMT in the airway of COPD patients is likely to be an important bridge between airway fibrosis and cancer development [8]. An analysis of nine prospective cohorts suggests that inhaled corticosteroids have a protective effect against lung cancer in COPD patients. Results showed that inhaled corticosteroids were correlated with a reduced risk of lung cancer in COPD patients, providing clinicians with guidance for lung cancer prevention in COPD patients [156]. However, further study is needed to determine whether inhaled corticosteroids have a protective effect against lung cancer in COPD through anti-EMT. These may suggest that EMT may be a potential pathway through which the therapeutic effects of inhaled corticosteroids may occur in COPD patients. This has important implications for treatment and public health policy. Because it recommends that inhaled corticosteroids or other drugs with similar effects be administered early in the natural course of COPD, this not only inhibits airway inflammation, but may also inhibit epithelial activation, EMT, and associated fibrosis and malignant consequences. Larger sample sizes are needed in the future to verify whether inhaled corticosteroids are treated with EMT in COPD by acting directly on the EMT.

### *N*-acetylcysteine (NAC)

The antioxidant *N*-acetylcysteine (NAC) is a mucolytic agent that is involved in the treatment of COPD as a mucolytic agent and has been shown to prevent exacerbation of COPD [157, 158]. COPD rat models demonstrated that *N*-acetylcysteine significantly reduced α-SMA level, collagen volume fraction, wall thickness/bronchiole diameter, and wall area/total bronchiole area (MA%) in COPD rats. Moreover, NAC inhibits the EMT process by inhibiting VWF/P38 MAPK signaling pathway. This suggested antioxidant *N*-acetylcysteine may contribute to EMT inhibition, thereby alleviating COPD pulmonary fibrosis. NAC could ameliorate COPD-induced pulmonary fibrosis by promoting immune response and inhibiting the EMT process via the VWF/p38 MAPK axis [15].

### Phosphodiesterase-4 (PDE4) inhibitors and statins

Phosphodiesterase 4 (PDE4) inactivates adenosine cyclophosphamide and guanosine cyclophosphamide and is the main PDE isoenzyme in cells involved in inflammatory airway diseases such as COPD. Roflumilast, an oral PDE4 inhibitor, has proven to reduce the rate of acute exacerbation rates and help improve mortality and quality of life in COPD patients [159, 160]. Studies have found that PDE4 inhibitors could affect lung architectural remodeling. In vivo studies indicate that roflumilast mitigated cigarette smoke-induced airspace enlargement and alleviated bleomycin-induced lung fibrotic and vascular remodeling in mice [161, 162]. Likewise, Roflumilast N-oxide (RNO) protected CSE-induced EMT in human bronchial epithelial cells (HBECs). It was shown that RNO inhibited the upregulation of mesenchymal cell markers (α-SMA, vimentin, and collagen type I) induced by CSE in HBECs. RNO also reversed the downregulation of epithelial markers (E-cadherin, ZO-1, and KRT5) in HBECs. Moreover, RNO reduced mesenchymal markers while increasing epithelial markers in primary human bronchial epithelial cells isolated from smokers and COPD patients’ small bronchi. In addition, RNO reduced the CSE-induced increase in TGF-β1 release and Smad3 and ERK1/2 phosphorylation [163].

Statins are commonly prescribed clinically to lower serum cholesterol. Retrospective studies [164–166] described statins improves survival in patients with lung cancer. However, whether statins’ effectiveness in lung cancer remains controversial [167, 168]. Nishikawa et al. [169] described statins suppress EMT and improve the prognosis of lung adenocarcinoma patients in a p53 mutation-dependent manner. Recently, population pharmaco-epidemiological evidence suggests that statin use reduces the risk of lung cancer in patients with COPD [170, 171]. Milara et al. [121] proved that RNO partly alleviated the CSE-induced EMT and simvastatin increases the ability of RNO to Inhibit CSE-induced EMT in HBEC in vitro. Moreover, PDE4 inhibitor and statin may act on different pathways involved in CSE-induced EMT, such as ROS, PI3K/Akt, GTP-Rac1, and nuclear β-catenin. Further preclinical and clinical trials of PDE4 inhibitors and statins will be required to determine their efficacy as a single or combined treatment of EMT in COPD.

### Urokinase-type plasminogen activator and urokinase-type plasminogen activator receptor (uPA and uPAR)

The binding of urokinase-type plasminogen activator (uPA) with urokinase-type plasminogen activator receptor (uPAR) is involved in the proteolytic activation of plasmin, which degrades fibrin and other ECM components, activating matrix metalloproteinases and promoting cell migration [172]. A retrospective study found that uPA and uPAR expression was significantly increased in pulmonary macrophages and alveolar wall cells from patients with COPD compared to the control, and that uPA expression was positively correlated with collagen levels [173]. Similarly, UPA and uPAR expression were increased in the airway epithelium of smokers and COPD patients compared with non-smokers. Moreover, in human small airway epithelial cell lines (HSAEpiCs), uPA and uPAR inhibition can block CSE-induced EMT by reversing the expression of E-cadherin and α-catenin and delaying the induction of N-cadherin and Vimentin [174, 175].

### Adipose-derived stem cell

Adipose-derived stem cells (ADSCs) are the most abundant stem cell type in adults. The transplantation of ADSCs by intravenous injection could reduce inflammatory cell infiltration, airway enlargement, and lung cell death in CS-exposure mice [176, 177]. In vitro studies, ADSC-conditioned medium culture effectively reversed CSE-induced decreased E-cadherin expression, increased Vimentin expression, and accelerated cell migration in A549 cells [178]. This suggests that ADSCs might be a potential target for EMT in CSE-induced COPD or lung cancer. However, future animal studies and clinical trials are needed to verify these findings.

### Ginsenoside Rg1

Ginsenoside Rg1 is the main active ingredient of Panax ginseng, having anti-inflammatory, antioxidant, and neuroprotective actions [179]. In CS-exposed COPD rats and CSE-exposed human bronchial epithelial (HBE) cells, Ginsenoside Rg1 alleviated CS or CSE-induced EMT via blocking the regulation of α-SMA and E-cadherin expression induced by CS or CSE. Additionally, ginsenoside Rg1 inhibited CSE-induced EMT through the TGF-β1/Smad pathway in HBE [108].

### Other EMT-targeted therapies in clinical applications

EMT has been increasingly recognized as an interesting target for the development of new therapeutic strategies. Particularly, multiple EMT-targeted therapies have emerged in oncology over the last decade. So far, reasonable strategies such as inhibition of EMT induction, reversal of EMT process, and strategic killing of cells undergoing EMT seem to be promising in controlling the occurrence of EMT [180].

Galunisertib (LY2157299), a TGFβ receptor 1 inhibitor, has been studied in clinical trials in various solid tumors [181–184]. Notably, Galunisertib has been reported to be significantly sensitive to enzalutamide treatment in prostate cancer by inhibiting TGF-β-mediated EMT process [185]. This suggests that the anticancer effect of Galunisertib may be partly attributable to its ability to inhibit EMT. Additionally, other target inhibitors such as COX 2 inhibitors and AXL inhibitors have been found to block the EMT induction. Celecoxib, a selective cyclooxygenase‑2 inhibitor, has synergistic anti-cancer effects in different cancer types and has been found to inhibit the EMT process in oral squamous cell carcinoma, hypopharyngeal ca, cancer and bladder cancer, etc. [186–189]. AXL is a tyrosine kinase receptor that has been reported as an oncogene in a range of cancers, including NSCLC [190]. Cabozantinib, a tyrosine kinase inhibitor that includes AXL, has been reported to reverse EMT-associated osimertinib resistance in NSCLC [191]. In addition, cabozantinib suppressed EMT-associated sunitinib resistance in renal cell carcinoma [192]. All-trans retinoic acid (ATRA) is a front-line treatment of acute promyelocytic leukemia and neuroblastoma [193, 194]. ATRA is now widely used in preclinical and clinical studies and has shown great anticancer potential in phase II and III clinical trials in patients with various cancers [180]. Specifically, ATRA has been shown to reverse the EMT process in breast cancer cells and hepatocarcinoma cells [195–197]. Compared with the prevention of EMT induction, a promising alternative strategy is to selectively target EMT-induced mesenchymal‐like cancer cells by therapeutically inhibiting the functions of EMT-specific markers. N-cadherin antagonist ADH-1 is the synthetic cyclic peptide (also known as CHAVC and Exherin). The biological effects of ADH-1 on tumors have been extensively investigated in various preclinical animal models and clinical trials [198]. It was found that ADH-1 treatment significantly enhanced the anti-tumor effects of chemotherapy in melanoma [199]. In addition to anti-tumorigenic drugs, anti-fibrosis drugs also have potential EMT targeting effects. Notably, the anti-fibrosis drugs pirfenidone and nintedanib, which are approved for the treatment of idiopathic pulmonary fibrosis (IPF), appear to work by inhibiting the TGF-β pathway [200, 201]. Pirfenidone has been reported to inhibit EMT in pulmonary fibrosis by regulating the Wnt/GSK-3β/β-catenin and TGF-β1/SMad2/3 signaling pathways [202]. Furthermore, nintedanib inhibited EMT by mediating the TGF-β/Smad pathway in A549 alveolar epithelial cells [203]. Collectively, all of these EMT-targeted therapies hold great promise in terms of translating into inhibiting the development of COPD and lung cancer. However, further preclinical experiments and clinical trials are required to validate the clinical benefits of EMT-targeted therapies in COPD.

## Conclusions

In recent years, EMT has become one of the research hotspots in the pathogenetic mechanism research of COPD. EMT is considered to be part of the pathophysiology of COPD-related fibrosis, remodeling, and malignant consequences. In this review, we summarized the effects of cigarette smoke on the pathogenesis of COPD, and focus on the cigarette smoke-induced EMT in COPD that occurs in the development of the latest clinical evidence. We reviewed the current research and treatment approaches for EMT in COPD. Therapies such as Inhaled corticosteroids (ICS) may offer EMT-targeting treatments that suppress airway inflammation, epithelial activation, EMT, and related fibrotic, and malignant consequences, offering a new idea for the use of ICS. Novel approaches to suppressing EMT formation or the associated inflammation are in development and represent an important therapeutic target. In conclusion, this review highlights the importance of understanding the molecular mechanisms of EMT in smoke-induced COPD, which is critical for identifying innovative therapies targeting EMT in COPD.

## Data Availability

Not applicable.

## Abbreviations

COPD: Chronic obstructive pulmonary disease
EMT: Epithelial–mesenchymal transition
TGF-β: Transforming growth factor-β
CS: Cigarette smoke
CSE: Cigarette smoke extract
DAMP: Damage-associated molecular patterns
PAMPS: Pathogen-associated molecular patterns
ROS: Reactive oxygen species
TLRs: Toll‑like receptors
NLRs: NOD‑like receptors
RNS: Reactive nitric oxide
ER: Endoplasmic reticulum
UPR: Unfolded protein response
FSP1: Fibroblast specific protein 1
β2M: β2-Microglobulin
MHC I: Major histocompatibility complex
TGF-β: Transforming growth factor-β
NF-kB: Nuclear factor-kappa B
NAC: *N*-Acetylcysteine
SABA: Short-acting Beta2-agonists
LABA: Long-acting Beta2-agonists
SAMAs: Short-acting antimuscarinics
LAMAs: Long-acting muscarinic antagonists
ICS: Inhaled corticosteroids
PDE4: Phosphodiesterase-4
uPA: Urokinase-type plasminogen activators
uPAR: Urokinase-type plasminogen activator receptor
ADSC: Adipose-derived stem cell
ADSC-CM: Adipose-derived stem cell-conditioned medium
